# Vagus nerve electroneurogram-based detection of acute kainic acid induced seizures

**DOI:** 10.3389/fnins.2024.1427308

**Published:** 2024-08-07

**Authors:** Elena Acedo Reina, Enrique Germany Morrison, Ayse S. Dereli, Elise Collard, Romain Raffoul, Antoine Nonclercq, Riëm El Tahry

**Affiliations:** ^1^Clinical Neuroscience, Institute of Neuroscience (IoNS), Université Catholique de Louvain, Brussels, Belgium; ^2^Walloon Excellence in Life Sciences and Biotechnology (WELBIO) Department, WEL Research Institute, Wavre, Belgium; ^3^BEAMS Department, Université Libre de Bruxelles, Brussels, Belgium; ^4^Department of Neurology, Center for Refractory Epilepsy, Cliniques Universitaires Saint-Luc, Brussels, Belgium

**Keywords:** vagus nerve electroneurogram, kainic acid, closed-loop VNS, seizure detection, autonomic nervous system, vagus nerve

## Abstract

Seizures produce autonomic symptoms, mainly sympathetic but also parasympathetic in origin. Within this context, the vagus nerve is a key player as it carries information from the different organs to the brain and vice versa. Hence, exploiting vagal neural traffic for seizure detection might be a promising tool to improve the efficacy of closed-loop Vagus Nerve Stimulation. This study developed a VENG detection algorithm that effectively detects seizures by emphasizing the loss of spontaneous rhythmicity associated with respiration in acute intrahippocampal Kainic Acid rat model. Among 20 induced seizures in six anesthetized rats, 13 were detected (sensitivity: 65%, accuracy: 92.86%), with a mean VENG-detection delay of 25.3 ± 13.5 s after EEG-based seizure onset. Despite variations in detection parameters, 7 out of 20 seizures exhibited no ictal VENG modifications and remained undetected. Statistical analysis highlighted a significant difference in Delta, Theta and Beta band evolution between detected and undetected seizures, in addition to variations in the magnitude of HR changes. Binomial logistic regression analysis confirmed that an increase in delta and theta band activity was associated with a decreased likelihood of seizure detection. This results suggest the possibility of distinct seizure spreading patterns between the two groups which may results in differential activation of the autonomic central network. Despite notable progress, limitations, particularly the absence of respiration recording, underscore areas for future exploration and refinement in closed-loop stimulation strategies for epilepsy management. This study constitutes the initial phase of a longitudinal investigation, which will subsequently involve reproducing these experiments in awake conditions with spontaneous recurrent seizures.

## 1 Introduction

Since its recognition as a viable therapy in 1988, Vagus Nerve Stimulation (VNS) has been employed to treat drug-resistant epilepsy, with over 125,000 patients implanted worldwide (Fetzer et al., 2021). Distinguished by its minimal invasiveness, VNS has demonstrated significant efficiency, with 45–60% of patients experiencing over 50% reduction in seizure frequency after a minimum treatment duration of 6 months (Toffa et al., 2020).

Initially, VNS utilized an open-loop system delivering electrical pulses based on a predetermined ON–OFF cycle. Featured in the device, magnets enable patients or caregivers to administer supplementary therapy manually. When magnet-activated stimulation was concurrently applied with VNS therapy, it demonstrated further clinical advantages, such as decreased seizure severity and an ability to terminate seizures (Boon, 2001; Morris, 2003), highlighting the therapeutic value of stimulating the vagus nerve proximal to seizure onset.

The introduction of the AspireSR VNS device in 2014 marked a significant advancement by employing a closed-loop system that delivers stimulation in response to ictal tachycardia, as 82% of seizures are characterized by heart rate (HR) increases during the pre-ictal or ictal phases according to literature (Devinsky, 2004; Eggleston et al., 2014). Challenges persist, despite the proven clinical benefits of a closed-loop system compared to traditional open-loop stimulation (Hamilton et al., 2018; Tzadok et al., 2019; Kawaji et al., 2020). Multicentric clinical studies referred to in the literature as “E-36” (Boon et al., 2015) and “E-37” (Fisher et al., 2016) highlighted a specificity concern, with only about 50% of seizures showing a significant ictal tachycardia (>20% HR increase) indicating significant variability in ictal HR changes among patients. Also, there is a concern about nonseizure-related detections ranging from 0.4 to 1.6 detections per hour at the maximum threshold of 70% and up to 6.6 and 6.9 for the minimum threshold of 20% (Boon et al., 2015) and 30% (Fisher et al., 2016), indicating a moderate specificity (Hampel et al., 2015). Hence, there is a need to increase the specificity of seizure detection strategies.

Electroencephalography (EEG) remains the gold standard for seizure detection in a clinical setting. It is utilized in Responsive Neurostimulation (RNS) therapy, which boasts a remarkable efficacy with a 75% median reduction in seizure frequency at 9 years (Nair et al., 2020). However, the high invasiveness of this therapy and its limited approval worldwide underscores the necessity for alternative, less invasive detection methods. Given the extensive autonomic dysfunctions induced by seizures, including changes in cardiac and respiratory patterns (Wannamaker, 1985; Devinsky, 2004; Sevcencu and Struijk, 2010), exploring biomarkers that integrate multi-organ information represents an promising innovative approach to seizure detection.

As the vagus nerve is accessed during electrode implantation for VNS and is the primary parasympathetic relay of the nervous system, recording the nerve using the same implanted electrode could contribute to the development of a novel method for seizure detection. In a study of systemic kainic acid injection in urethane anesthetized rats, the vagus nerve and the cervical sympathetic ganglion were recorded with invasive linear array electrodes to determine seizure-related modifications in the autonomic nervous system (ANS) (Naggar et al., 2022). They found that in the severe outcomes (self-terminating seizures with severe bradyarrhythmia and seizure-related death), ANS activity increased during seizures until it caused a drastic HR reduction (>50%), in which case seizure and ANS activity decreased dramatically (Naggar et al., 2022). Specifically, parasympathetic nerve activity was maintained during seizures, resulting in death, and the persistence of sympathetic tone was important for survival during the late vulnerable period in self-terminating seizures (Devinsky et al., 2016; Naggar et al., 2022). Hence, patterns of vagus nerve activity could give indications of seizure characteristics and therefore be proposed as a biomarker for seizure detection. Indeed, prior studies have successfully extracted a cardiac-related profile from the vagus nerve electroneurogram (VENG) as a biomarker to detect pentylenetetrazol (PTZ) induced seizures in rats (Harreby et al., 2011). However, none of these studies recorded the vagus nerve with non-invasive electrodes, with the final goal of extracting an ictal VENG pattern for seizure detection. Also, in addition to HR changes, seizures are also characterized by respiratory dysrhythmias and, in severe cases, hypopneas and apneas (Nashef et al., 1996; Seyal and Bateman, 2009; Sowers et al., 2013; Stewart, 2018; Sivathamboo et al., 2020; Faingold and Feng, 2023). In a study investigating VNS as a potential treatment for refractory hypertension, respiratory components were successfully extracted from the VENG, as a potential biomarker to differentiate between exercise-induced increase in blood pressure and pathological hypertension (Sevcencu et al., 2018). Therefore, respiratory-based VENG activity is measurable and can be proposed as a novel way of detecting seizures.

Our previous work introduced non-invasive monitoring of the vagus nerve to detect systemic PTZ-induced generalized seizures in rats, using a microcuff electrode around the left cervical portion of the vagus nerve. This VENG-based detection showed independence from movement artifacts and outperformed heart-rate-based seizure detection algorithms (Stumpp et al., 2020, 2021). Nonetheless, PTZ’s limitations, including its tendency to induce hyperexcitability and interictal spikes shortly after administration, prompted our focus on acute intrahippocampal kainic acid-induced seizures. This model mimics human temporal lobe epilepsy (TLE) characteristics, including associated autonomic dysfunctions like respiratory abnormalities, apnea, and blood pressure and HR changes, thus reinforcing the relevance of this model for advancing seizure detection research (Wannamaker, 1985; Lévesque and Avoli, 2013).

This investigation aims to refine our seizure detection algorithm by exploring respiratory-derived ictal VENG modifications in an anesthetized acute intrahippocampal KA model using implantable cuff electrodes. This study will be extended to awake conditions to confirm the detection feasibility for potential future closed-loop stimulation strategies.

## 2 Methods

### 2.1 Animals

Six male Wistar rats (Local breeding facility, UCLouvain, Belgium), with an average weight of 296.8 ± 50.2 g, were housed in a 12 h day/night cycle in a temperature-and humidity-controlled environment with ‘*ad libitum*’ access to food and water. The protocol has been approved by the Animal Experimental Ethical Committee of UCLouvain University and the Brussels environment committee under the reference 2022/UCL/MD/066.

### 2.2 Surgical procedure

Initially, all animals were anesthetized using a combination of ketamine (100 mg/kg) and xylazine (7 mg/kg) administered intraperitoneally. To maintain anesthesia, half the initial dose was administered as soon as a withdrawal reflex was observed. On average, each animal received two maintenance injections, totaling 200 mg/kg of ketamine and 14 mg/kg of xylazine.

A rectal probe was inserted to monitor and control body temperature, and a heating pad was used to maintain a constant temperature of 37.5°C. The animals were then securely positioned in a stereotaxic frame (David Kopf Instruments, Tujunga, USA) for the implantation procedure. Custom-made epidural electrodes, consisting of a 4.8 mm stainless-steel screw (Bilaney, Germany), were implanted at specific coordinates relative to the Bregma: for the frontal electrode (+) [anteroposterior (AP) +2 mm, mesolateral (ML) ±3 mm], reference electrode (Ref) [AP +6 mm, ML ±0 mm], and ground electrode (GND) [AP −5 mm, ML +3 mm] in the parietal region (Medlej et al., 2019). The differential EEG recording was obtained between the frontal (+) and parietal (Ref) electrodes.

An additional procedure involved drilling a hole into the right hippocampus for kainic acid (KA) injection with the coordinates [Right Hippocampus (RH) AP −5.6 mm, ML −4.5 mm]. Following this, the left vagus nerve was exposed at its cervical portion, and a tripolar micro-cuff electrode (Microprobes, Gaithersburg, USA) was carefully implanted around the nerve for vagus nerve recording. To ensure the electrode remained in place, the sternomastoid and sternohyoid muscles were sutured over it. The implanted cuff electrodes have specific dimensions: 300 μm inner diameter, 100 μm contact width, and 4 mm contact spacing. The recording setup was designed to reject common noise, with signal amplitudes from two channels being differentially analyzed. In cases of signal amplitude disparity between the two channels, the channel with the amplitude closest to the mean was retained. Electrode impedance was measured before the surgery, with acceptable levels defined as lower than 30 kΩ. Several experiments were previously performed to confirm the genuine origin of the VENG recorded signals with tripolar microcuff electrodes (Stumpp et al., 2020; Smets et al., 2021).

Finally, following Eindhoven’s lead II reference, three lab-made tungsten electrocardiogram (ECG) electrodes were implanted subcutaneously to monitor cardiac activity.

### 2.3 Experimental procedure

Post-surgery, each animal was carefully placed within a Faraday cage, with their head fixed in the stereotaxic frame to ensure minimal movement and interference during the recording phase. For the administration of KA, a 25G Hamilton microliter syringe, preloaded with a KA solution (0.4 μg/0.2 μL; Hello Bio, Princeton, USA) as per Bragin et al. (1999) was mounted to the stereotaxic frame. This setup allowed for precise delivery of KA into the right hippocampus, targeting the coordinates previously established during the surgical procedure.

The electrophysiological data collection encompassed EEG, ECG, and VENG recordings, providing a comprehensive overview of the induced seizure activity and its effects. Initially, a 20 min baseline recording was captured to establish the background activity levels for each animal. Following this, the KA solution was injected into the hippocampus at a controlled rate of 0.1 μL/min (DV: 4.5 mm deep), aiming to induce acute seizure episodes. This injection phase was immediately succeeded by a 20 min ictal recording session to capture the seizure manifestations. After the ictal recording, the epileptic activity was stopped by a mixture of Diazepam (20 mg/kg) and Ketamine (50 mg/kg), administered intraperitoneally, as recommended by Martin and Kapur (2008) and Vermoesen et al. (2010). This led to a final 20 min post-ictal recording period. The experimental sequence concluded with the euthanasia of the animals through CO_2_ asphyxiation, ensuring a painless and ethical end to their participation in the study.

### 2.4 Data acquisition and filtering

For data acquisition in this study, a suite of custom-made amplifiers was employed, including two amplifiers for the EEG, one for the ECG, and two for the VENG. Each type of signal was subjected to specific band-pass filtering (hardware and software) to ensure the integrity and quality of the recorded signals. ECG signals were filtered between 1 and 70 Hz, EEG signals between 0.5 and 70 Hz to cover key EEG bands, while VENG filtering was between 300 Hz and 3 kHz is based on nerve fiber conduction velocities to capture relevant physiological signals accurately (Lee, 2009; Mollet et al., 2013; Qing et al., 2018). This filtering process was crucial for isolating the relevant frequency bands and minimizing noise in the recorded data.

Signal digitization was conducted at different sampling frequencies tailored to each signal type to capture the necessary temporal resolution and detail: EEG signals at 250 Hz, ECG at 40 kHz, and VENG at 80 kHz. This approach allowed for comprehensive data analysis, effectively capturing the intricacies of each signal type.

Data acquisition was facilitated by a USB-6212 multifunction I/O device (National Instruments, Austin, USA). The recorded data were then processed and stored using a custom-developed application in Matlab R2022a (MathWorks, Natick, USA). This setup ensured that the data were not only captured with high fidelity but also organized and accessible for subsequent analysis, laying the groundwork for the detailed investigation of the induced seizures and their physiological manifestations. The acute monitoring system and its physiological amplifiers were previously validated (Stumpp et al., 2020; Smets et al., 2021).

### 2.5 Data analysis

This section details the methodologies for analyzing the various physiological signals recorded during the experiment. The analysis includes determining seizure onset and duration from EEG, EEG band power evolution, ECG-derived HR and respiration activity, and VENG-based seizure detection derived from respiratory pattern changes. Each sub-section provides a step-by-step description of the signal processing and analysis techniques used.

#### 2.5.1 EEG seizure onset and duration

Seizure events were identified by periods of low-voltage, high-frequency (>5 Hz) rhythmic spiking activity, evolving into high-voltage low-frequency spike-and-wave discharges lasting at least 10 s. The end of the seizure was defined as the last spike observed on the EEG (Van Nieuwenhuyse et al., 2015). The onset and offset of these seizure events determined the total seizure duration.

#### 2.5.2 Individual ictal EEG band power evolution calculation

Spectral power was computed through Fourier transform over an 8 s sliding window with 50% overlap. A pre-ictal window, mirroring the ictal duration, was selected for normalization against the mean spectral power. This normalized spectral power, smoothed with a 5-sample moving average window, was expressed as a percentage change relative to the pre-ictal state.

#### 2.5.3 ECG-derived ictal heart rate changes

Instantaneous HR was determined by measuring the interval between successive R peaks in the QRS complex. This approach allowed for calculating the mean instantaneous HR over 10 s intervals. We compared the HR at seizure onset against the maximum and minimum HR recorded during the seizure to ascertain the extent of HR variation (peak increase/decrease). Additionally, we evaluated the mean HR increase or decrease during the seizure by comparing the average instantaneous HR within the seizure window to the baseline pre-ictal period, which was matched in duration to the ictal window (average HR increase/decrease). This comparative analysis facilitated a detailed understanding of the cardiac responses associated with seizure activity.

#### 2.5.4 ECG-derived respiration calculation

Respiration frequency was estimated through the amplitude modulation of R peaks within the ECG’s QRS complex, reflecting the mechanical and electrophysiological effects of inspiration and expiration. This method provides an indirect measure of respiratory activity during the experimental protocol (Charlton et al., 2018).

#### 2.5.5 VENG-based seizure detection

**Figure 1:**
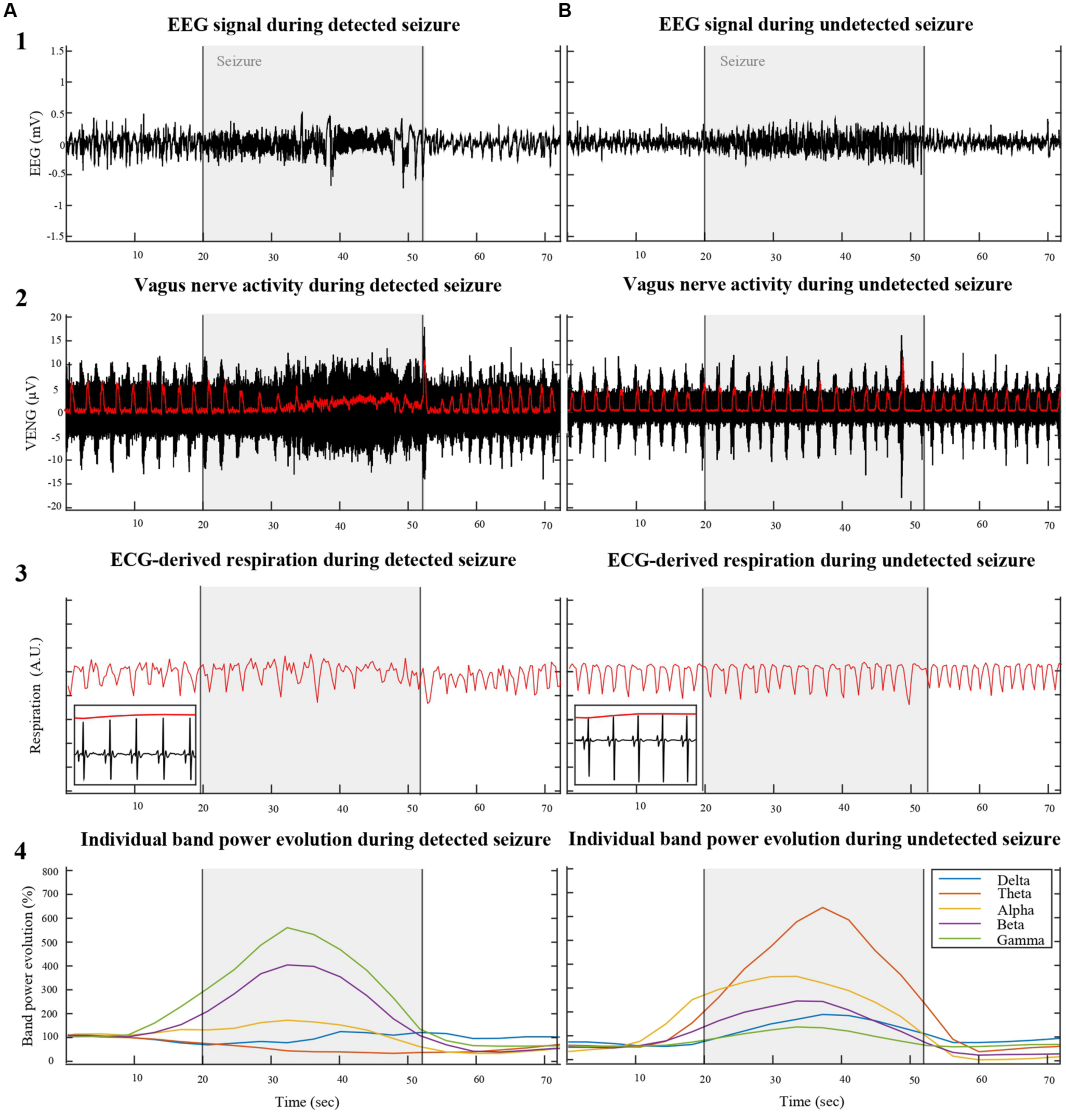
Illustrates a comparative analysis of physiological parameters between VENG-detected **(A)** and undetected **(B)** seizures induced by KA injection into the right hippocampus of two different rats (RAT3 and 5), weighing 215 g and 306 g, respectively. The top row displays the EEG signal across three phases: pre-ictal, ictal, and post-ictal. The second row depicts vagus nerve activity during the same periods, with respiration-related VENG bursts (indicated by the red line in row 2) synchronizing with respiration derived from modulation QRS complexes of the ECG during in/expiration (red line in row 3). The bottom row represents the individual EEG band spectrum evolution during the recorded seizures, normalized by the pre-ictal period, emphasizing the heightened activity in each band during the ictal period.

**Figure 2:**
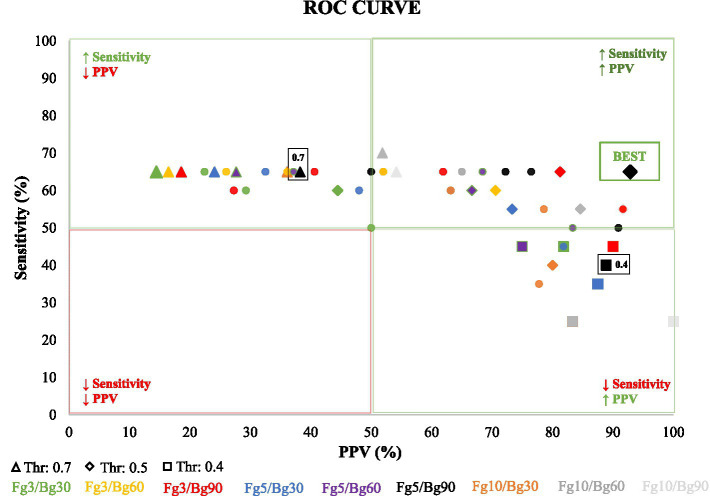
Illustrates Receiver Operating Characteristic (ROC) curves, depicting the predictive performance of various detection parameters, including the detection threshold and Foreground/Background (Fg/Bg) window length. The *x*-axis represents the accuracy or positive predictive value (PPV), while the *y*-axis represents the true positive rate (TPR), also known as sensitivity. Each curve corresponds to a unique combination of Fg/Bg window sizes, with points along the curves indicating different detection thresholds, ranging from 0.70 (on the left) to 0.40 (on the right), with a step decrement of 0.05. Optimal detection parameters, determined by maximal sensitivity and positive predictive value, are highlighted at a detection threshold of 0.5 and an Fg/Bg length of 5/90 s.

**Figure 3:**
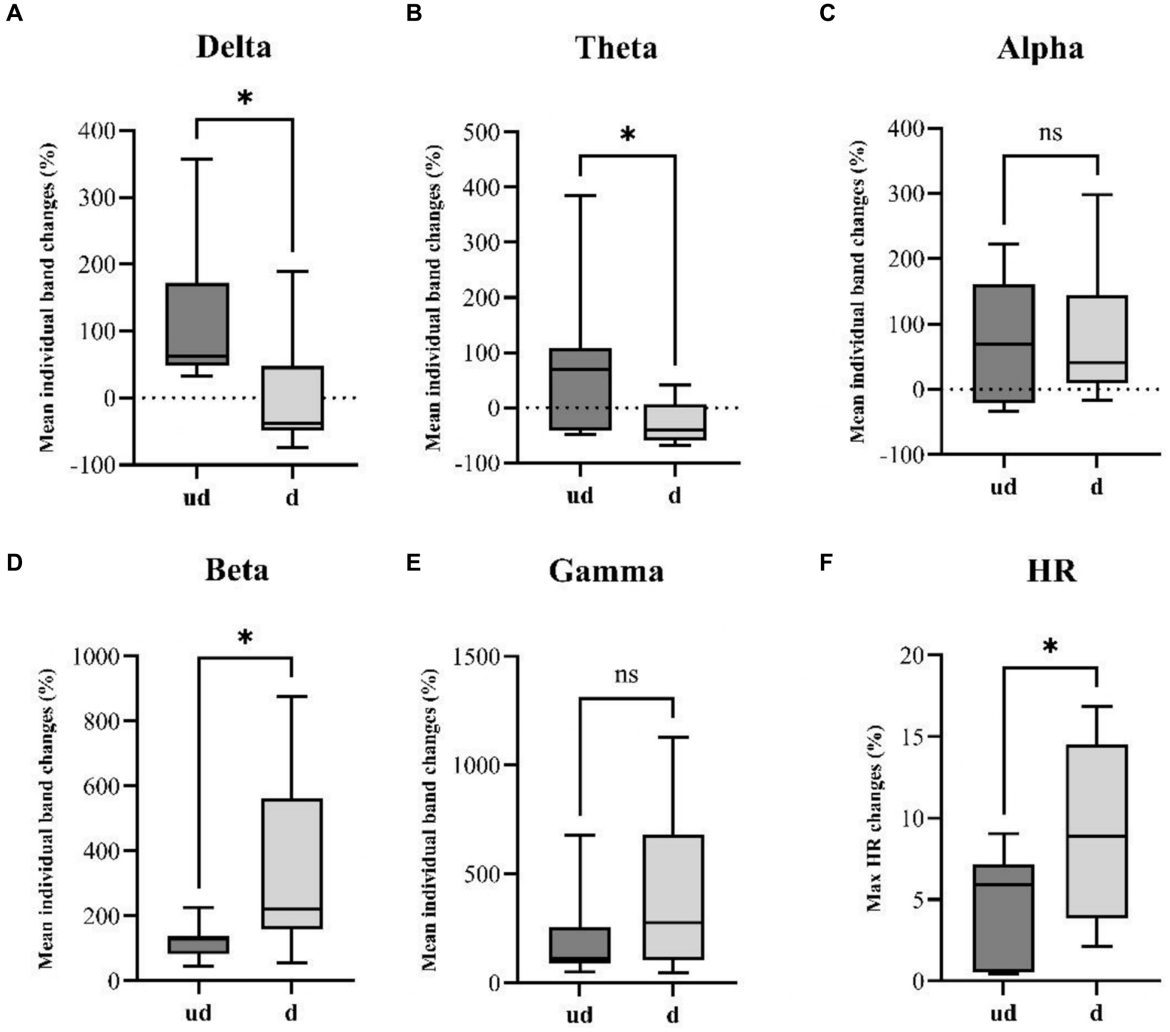
Illustrates a comparative analysis of seizure spread and HR changes between undetected seizures (ud, *n* = 7) and detected seizures (d, *n* = 13). Boxplots resulting from the Mann–Whitney U-test reveal a significant increase in panel **(A)** delta (*p*-value: 0.0136) and **(B)** theta (*p*-value: 0.0236) band power in undetected seizures. Conversely, detected seizures exhibit a significantly higher increase in faster **(D)** beta band (*p*-value: 0.0297) power. Additionally, unpaired t-tests reveal a significantly steeper HR changes **(F)** in detected seizures compared to undetected seizures. While no significant differences were observed in panel **(C)** Alpha and **(E)** Gamma bands. *p<0.05.

We aimed to detect acute seizure-related changes by focusing on alterations in respiration-related VENG bursts. The integrity of the VENG signal was assessed by evaluating the synchronization of these bursts with the frequency and phase of ECG-derived respiration (Figures 1–3), as previously implemented by Charlton et al. (2018), Patros et al. (2022), and Sevcencu et al. (2018). First, upper and lower-bound envelopes were computed to determine the peak-to-peak values, thereby measuring the distance between these envelopes. This peak-to-peak amplitude signal was subsequently smoothed with a 100 ms averaging window to minimize noise and stabilize the signal for analysis.

Following smoothing, a Fast Fourier Transform (FFT) was applied to isolate the primary low-frequency components that match the overall main respiratory frequency (*R_f_*) within the VENG envelope signal. Centered on this identified respiratory frequency a narrow band-pass filter as shown in Equation (1), was employed to track the amplitude of respiratory components within the surroundings of *R_f_* over time. As respiratory frequency deviates from the narrow band range, the amplitude of the narrowed bandpass filtered signal decreases.

(1) $$\mathrm{Low}\;\mathrm{cutoff}\;\mathrm{frequency}=\frac{R_{f}}{2}\;\mathrm{and}\;\mathrm{High}\;\mathrm{cutoff}\;\mathrm{frequency}=3R_{f}$$

Our seizure detection algorithm calculates a ratio using two distinct moving averaging windows: a smaller foreground (Fg) window and a larger background (Bg) window. By methodically sliding these windows across the signal and comparing the ratio to a given threshold Equation (2), abrupt respiratory changes can be identified.

(2) $$\frac{F_{g}}{B_{g}}<th$$

Normally, the Fg/Bg ratio is expected to hover around 1, indicating stable respiratory amplitude under non-seizure conditions. However, seizures prompt abrupt changes in respiratory frequency, leading to a notable decrease in this ratio. To optimize detection sensitivity, we experimented with various sizes for the Fg (3, 5, and 10 s) and Bg (30, 60, and 90 s) windows, and the thresholds for this ratio were methodically adjusted between 0.4 and 0.7, in increments of 0.05. This process allowed us to detect abrupt deviations in respiratory frequency, indicative of seizure activity, thus highlighting the intricate relationship between respiratory dynamics and seizure occurrences.

### 2.6 Statistical analysis

The statistical framework of our study was designed to evaluate the efficacy of our seizure detection algorithm. The sensitivity of seizure detection was determined by calculating the ratio of true positive detections to the total number of seizures observed. Additionally, the positive predictive value (PPV), which provides insight into the accuracy of the seizure detections, was derived by dividing the number of true positive detections by the sum of true positive and false positive detections.

To further refine our understanding of the algorithm’s performance, Receiver Operating Characteristic (ROC) curves were constructed. These curves plot the sensitivity of the seizure detection against the PPV for various combinations of foreground/background (Fg/Bg) window lengths and detection thresholds. Sensitivity represents the proportion of true positives correctly identified by the algorithm, while PPV indicates the proportion of detected events that are true positives. The ROC curves are a critical tool for assessing which combination of these parameters yields the most effective detection strategy.

All statistical analyses were performed using GraphPad Prism version 10.1.2 (GraphPad Software, Boston, MA, USA) and JASP 0.18.3.0. Seizures were categorized into two groups: detected seizures (d) and undetected seizures (ud). Normality of data was assessed using the Shapiro–Wilk Test. Parametric and non-parametric unpaired t-tests and Mann–Whitney U tests (MWU) were conducted to compare mean band power evolution between groups, with significance determined at *p* < 0.05. Data were expressed as mean ± SD. Logistic regression analyses were performed only for physiological parameters that showed a significant difference in parametric and non-parametric t-tests. Binomial logistic regressions were employed to ascertain whether the likelihood of seizure detection significantly correlates with various predictive variables, including the evolution of individual Delta, Theta and Beta bands, and Maximum HR changes (Schober and Vetter, 2021). Logistic regression outcomes were reported in terms of log odds ratios (OR), 95% confidence intervals (CI), and *p*-values, with a *p*-value of less than 0.05 denoting a statistically significant correlation between the detection outcomes and the predictive variables under investigation. Additionally, regression coefficient, standard error, *R*^2^ de McFadden and area under the curve (AUC) were added in the table. Our study implemented uniform subject cohorts, standardized anesthesia protocols, and controlled environmental conditions to mitigate potential confounders in logistic regression analyses.

## 3 Results

### 3.1 Characterization of the induced seizures

In our study, we observed a range of two to five KA-induced seizures per animal (*n* = 6), with a total of 20 recorded seizures (Table 1). The average duration of these seizures was 53.1 ± 19.1 s. Remarkably, during the entirety of the ictal recordings, no motor manifestations were detected, attributed to the constant state of anesthesia under which the seizures were induced. Notably, two seizures were triggered immediately following the injection of kainic acid. In contrast, the remaining 18 seizures manifested after a mean delay of 8.8 ± 4 min post-injection, with seizures occurring at an average interval of 4.8 ± 3.2 min.

**Table 1:** Summarizes seizure characteristics per animal, including the count of seizures (No sz), average duration, mean HR changes throughout the seizure, maximum HR changes during the seizure, the number of seizures detected by the algorithm (using Fg5/Bg90, Threshold: 0.5), the mean delay between EEG seizure onset and VENG seizure detection onset, and specificity/positive predictive value.

	No. sz (EEG)	Avg. duration (s)	Mean HR changes (%)	Max HR changes (%)	No. detected sz	Mean delay (s)	Sensitivity (%)	PPV (%)
RAT 1	5	52.9 ± 19.3	3.0 ± 1.6	6.8 ± 2.8	5	14.6 ± 4.6	100	100
RAT 2	3	57.13 ± 32.1	−0.7 ± 0.8	−3.1 ± 0.8	3	16.2 ± 2.7	100	100
RAT 3	3	31.2 ± 0.9	−3.5 ± 0.6	−13.6 ± 2.7	3	18.4 ± 3.4	100	100
RAT 4	4	70.7 ± 8.8	−4.5 ± 4.0	−12.0 ± 5.6	2	48.4 ± 18.2	50	100
RAT 5	3	43.4 ± 10.3	−0.2 ± 0.2	−0.6 ± 0.1	0	–	0	0
RAT 6	2	60.9 ± 2.3	−0.9 ± 1.1	−4.9 ± 5.9	0	–	0	0

When examining the EEG band activity for all seizures collectively, we noted a modest elevation in the delta and theta frequency ranges, with delta activity increasing by 43.6 ± 107.5% and theta by 18 ± 103%. However, the most pronounced changes were observed in the faster EEG frequency bands: alpha band activity surged by 91.7 ± 93.1%, beta by 240.4 ± 235.93%, and gamma by 286.3 ± 318.3%. This differential increase in EEG band activity underscores the varied neurophysiological impact of KA-induced seizures, highlighting significant alterations in brain activity, particularly within the higher frequency bands.

### 3.2 Heart rate during acute KA-induced seizures

The primary HR change outcome was ictal bradycardia in five out of six rats (RAT2-6). During the ictal phase, the mean average HR across all seizures in all rats decreased by 2.1 ± 2.3%, and the mean peak had a more pronounced decrease of 7.6 ± 6% compared to pre-ictal phase. These findings are detailed individually for each rat in Table 1.

The remaining rat (1/6) demonstrated ictal tachycardia, characterized by a mean average HR increase of 3 ± 1.6% and a mean peak increase of 6.8 ± 2.8% relative to the pre-ictal HR stage.

### 3.3 VENG during acute KA-induced seizures

Characterization of ictal modification of VENG signal revealed that seizures could be categorically divided into two distinct groups. The first group of 13 seizures, with a depicted representation in Figure 1A, was characterized by a noticeable disruption in the rhythmicity of respiration-related VENG bursts during seizures. The majority of this group (10 out of 13 seizures) exhibited a reduction in the mean amplitude during seizures, averaging 5.04 ± 3.44 μV. Specifically, a subset of these seizures (3 out of 13) was marked by an increase in the mean VENG signal amplitude, averaging 56.23 ± 41.17 μV. The second group, as illustrated in Figure 1B, was distinguished by an absence of discernible changes in VENG activity in response to the seizures, both in terms of amplitude and rhythmicity related to respiration in the signal.

This differential response in VENG signal amplitude and rhythmicity to KA-induced seizures provides valuable insights into the variability of autonomic nervous system reactions among individual subjects, potentially contributing to a more nuanced understanding of seizure dynamics and their impact on vagus nerve activity. Furthermore, these changes in amplitude and rhythmicity within the VENG signal were leveraged by the detection algorithm, confirming that 13 out of 20 seizures exhibit alterations, while the remaining 7 do not.

### 3.4 VENG-based seizure detection

In our study, a comprehensive approach was adopted to refine the parameters for optimal VENG-based seizure detection across the six rats (RAT1-6) involved in ictal recordings. We explored nine distinct combinations of foreground/background (Fg/Bg) window lengths, alongside seven detection thresholds ranging from 0.4 to 0.7, incremented by 0.05. This comprehensive analysis aids in identifying the most effective parameters for optimizing the algorithm’s performance in seizure detection.

For each Fg/Bg window combination, a Receiver Operating Characteristic (ROC) curve was generated by evaluating the sensitivity and Positive Predictive Value (PPV) at each threshold level (illustrated in Figure 2). Each curve symbolizes a specific Fg/Bg pairing, with individual points along the curve representing different detection thresholds. It was observed that higher thresholds led to less restrictive detection criteria, compromising accuracy. The thresholds were arranged such that they increased from right to left along the curve.

A notable observation from the black curve (Figure 2, Fg5/Bg90) reveals that the first point on the left (threshold: 0.7) achieved the highest sensitivity (65%) but the lowest accuracy (38.9%), indicating an overly permissive threshold. Conversely, the second highlighted point (threshold: 0.5) was identified as the optimal balance between sensitivity and accuracy, successfully detecting 13 out of 20 seizures (sensitivity: 65%) with only one false detection (PPV: 92.8%). The lowest tested threshold (0.4) resulted in the detection of 8 out of 20 seizures (sensitivity: 34.8%), accompanied by one false detection (PPV: 88.9%). Following the ROC curve analysis, the optimal parameters was set at Fg5/Bg90 and the detection threshold to 0.5. With these parameters, the VENG algorithm managed to detect 13 out of 20 seizures, recording a mean delay of 25.3 ± 13.5 s between the EEG onset and VENG detection onset (Table 1).

### 3.5 Comparative analysis of seizure spread through ictal band power evolution and HR changes between undetected and detected seizures

We compare the mean ictal band power evolution for each individual band to investigate potential differences in seizure spread between the two groups. The statistical analysis revealed a significant difference in the evolution of the Delta band (*p*-value: 0.0136), with undetected seizures exhibiting a mean increase of 117.6 ± 116% compared to the detected group with a mean change of 0 ± 79.6% (Figure 3). Similarly, significant differences were found in the Theta band (*p*-value: 0.0236), where the undetected group displayed a mean increase of 83.6 ± 145.6%, contrasting with the detected group with a mean decrease of −28.4 ± 38.2% (Figure 3). Additionally, in the faster Beta band, a significant difference in band power evolution was observed (*p*-value: 0.0297), indicating faster activity in the detected group (334.9 ± 263.4%) compared to the undetected group (122.4 ± 56.7%) (Figure 3). Furthermore, unpaired t-tests revealed a significant difference in the ictal HR changes between the two groups, with the detected group showing an absolute mean change of 9.03 ± 5.5% contrasted with the undetected group which showed an absolute HR change of 4.42 ± 3.7% (Figure 3).

The observed variations in individual EEG band power evolution between detected and undetected seizures suggest the possibility of distinct seizure spreading patterns between the two groups. This may result in differential activation of the autonomic central network. This observation is further supported by the differential HR changes observed between the two groups, indicating potential differences in the underlying mechanisms of seizure propagation.

### 3.6 Correlation between physiological parameters and VENG-based seizure detection efficacy

We conducted binomial logistic regression analyses to explore which physiological parameters affect VENG signal-based seizure detection. These focused on examining the associations between detection outcomes and several predictive variables, such as the evolution of individual EEG band power and average HR changes (ictal versus pre-ictal periods).

The binomial logistic regression revealed a nuanced interplay between VENG-based seizure detection and EEG band power evolution. Notably, an increase in theta band activity was associated with a decreased likelihood of seizure detection ([OR]: 0.976, 95% [CI]: [−0.047;-0.001], *p*-value: 0.038*), suggesting an impact of theta band fluctuations on the efficacy of seizure detection (Table 2). A similar trend was observed with delta band activity, although the correlation approached the statistical significance threshold (OR: 0.986, CI: [−0.029;0], *p*-value: 0.051) (Table 2). Conversely, an increase in beta band activity showed a tentative correlation with enhanced seizure detection (OR: 1.013, CI: [−0.003;0.029], *p*-value: 0.099), albeit not reaching statistical significance (Table 2).

**Table 2:** Presents the outcomes of logistic regression analyses, displaying the regression coefficients along with their corresponding error terms, the odds ratios with their 95% confidence intervals, Wald statistic-derived *p*-values, McFadden’s *R*^2^, and the area under the Receiver Operating Characteristic (ROC) curve.

	Coefficient	Error	OR	*p*-value	95% CI		McFadden *R*^2^	AUC
					Lower bound	Upper bound		
Delta	−0.015	0.007	0.986	0.051	−0.029	0	0.246	0.835
Theta	−0.024	0.012	0.976	0.038^*^	−0.047	−0.001	0.303	0.813
Beta	0.013	0.008	1.013	0.099	−0.003	0.029	0.261	0.802
HR	0.222	0.129	1.248	0.086	−0.032	0.475	0.158	0.736

*p<0.05.

Furthermore, the logistic regression analysis highlighted a correlation between HR changes and seizure detection, indicating that a more pronounced HR change is associated with a higher likelihood of detecting a seizure (OR: 1.248, CI: [−0.032;0.475], *p*-value: 0.086) (Table 2).

## 4 Discussion

This study demonstrates the feasibility of seizure detection based on respiratory-derived ictal VENG modifications using an implantable cuff electrode in an acute intrahippocampal KA model. Our algorithm successfully identified 13 out of 20 seizures with an accuracy of 92.9% and an average delay of 25.3 ± 13.5 s. However, 7 out of 20 seizures remained undetected, exhibiting distinct EEG characteristics and autonomic symptoms, suggesting potential differences in seizure spread and involvement of the autonomic central network.

Respiratory modulation of the vagus nerve activity is well established in the literature in both animal models and human studies. Previous research using a porcine model has shown that vagal recordings with cuff electrodes exhibit morphological similarities within the neural activity profile with respect to actual respiratory patterns, in both duration and frequency (Sevcencu et al., 2018). Further, studies involving awake human subjects have utilized intraneural recordings from the left vagus nerve, revealing modulations attributed to both cardiac and respiratory functions (Patros et al., 2022). Notably, respiratory modulation was observed to be more substantial (45%) compared to cardiac modulation (23%), suggesting a stronger respiratory influence on vagus nerve activity (Patros et al., 2022). In the current study, the baseline recordings showed that ECG-derived respiratory activity aligned well with the respiratory-related vagus nerve bursts, supporting previous findings and validating our setup (Charlton et al., 2018).

Our results suggest an ictal disappearance of spontaneous vagus nerve bursts related to respiration. This is potentially explained by well-recognized respiratory arrhythmia observed during seizures. In humans, seizures can be characterized by hypopneas (Bateman et al., 2008) and, in severe cases, obstructive apnea and respiratory arrest, leading to sudden death (Nashef et al., 1996; Seyal and Bateman, 2009; Sowers et al., 2013; Stewart, 2018; Sivathamboo et al., 2020; Faingold and Feng, 2023). Similarly, animal studies on KA rats have shown seizure-induced suppression of phrenic nerve activity (Ashraf et al., 2021), which is the only motor nerve innervating the diaphragm, hence a potential explanation for reduced respiratory frequency. In another study using an acute kainic acid rat model, respiratory efforts were observed during bradycardic seizures (Naggar et al., 2022). These respiratory efforts, characterized by gasps, were later explained by airway obstruction. This aligns with the emerging consensus that seizures can lead to obstructive apnea (Nakase et al., 2016; Stewart et al., 2017; Budde et al., 2018; Jefferys et al., 2019), which could, in severe cases, be followed by bradycardia, terminal apnea, and eventually death (Nakase et al., 2016; Budde et al., 2018). These autonomic alterations result in an increased flow of information through the vagus nerve, wherein bidirectional transmission can either increase or decrease signal amplitude. The modulation of rhythmicity may stem from respiratory irregularities disrupting respiration-related bursts or heightened information transit, masking these bursts. While our experimental setup did not directly measure apneas, the disturbances observed in respiratory-derived VENG signals during seizures could serve as potential biomarkers for seizure detection.

A previous investigation has explored a seizure detection algorithm based on VENG activity in the PTZ model focusing on cardiac-related changes (Harreby et al., 2011). Their method involved identifying variations in the extracted cardiac-related profile (CrVENG), obtained through R-peak triggered averaging of VENG energy. Notably, all seizures were detected with a mean delay of 103 ± 51 s before the onset of the tonic–clonic phase (Harreby et al., 2011). However, this study used hook electrodes, which are unsuitable for human application. Additionally, this algorithm exclusively relied on cardiac-related changes without exploiting all the potential of VENG detection associated with other non-cardiac physiological changes.

A more recent algorithm adopted a spike detection approach, quantifying VENG activity by analyzing extra-neural spikes or Compound Action Potentials (CAPs) (Stumpp et al., 2021). This method involves computing a detection metric by averaging spike frequency and amplitude and determining the ratio between two sliding Foreground (Fg) and Background (Bg) windows. All seizures were detected with 100% sensitivity and specificity, with 3 out of 6 seizures identified during the early stage, exhibiting a mean delay of 426 ± 126 s (7.10 ± 2.1 min) before the tonic–clonic stage. The remaining 3 out of 6 seizures were detected with a mean delay of 9.6 ± 5.4 s after the onset of the tonic–clonic stage. Analysis of spiking activity in the vagus nerve has become the focus of a growing number of studies with diverse objectives. Most studies have explored vagus nerve spiking activity in non-epileptic related diseases such as inflammatory response (Zanos et al., 2018), intestinal/gastric extension, or hypoxia (McCallum et al., 2017). This study provides a novel application of spiking analysis in the context of epilepsy.

By optimizing our new detection algorithm’s parameters, including varying Fg/Bg window lengths and setting detection thresholds (0.4–0.7 with increments of 0.05), we identified an optimal setup with an Fg/Bg window length of 5/90 s, coupled with a threshold of 0.5. This configuration allowed for detecting 13 out of 20 seizures, with a mean delay post-ictal onset of 25.3 ± 13.5 s, verified against concurrent scalp EEG recordings. However, it is noteworthy that RAT4 exhibited an extended detection time latency, with 2 out of 4 seizures detected at a mean delay of 48.36 ± 18.17 s. In contrast, the 11 seizures detected in the first three rats (RAT1-3) demonstrated a detection delay of 16.09 ± 2.67 s. Moreover, despite variations in algorithm parameters, 7 out of 20 seizures exhibited no ictal VENG modifications and remained undetected.

Our investigation explored the hypothesis that detected seizures might exhibit higher severity than undetected seizures, potentially explaining the absence of autonomic changes in the latter group. Since the conventional Racine scale is inadequate for assessing seizure severity due to the absence of clinical manifestations under anesthesia (Racine, 1972; Lüttjohann et al., 2009), we chose to explore individual band power evolution to identify potential differences in EEG characteristics that could indicate different seizure spread patterns or severity. Grouping all seizures together revealed a notable increase in gamma and beta bands during seizures, with detected seizures showing faster EEG activity. Binomial logistic regression further confirmed that the detection appeared to be correlated with a steeper increase in beta (OR: 1.013, CI: [−0.003; 0.029], *p*-value: 0.099), and a smaller increase in theta ([OR]: 0.976, 95% [CI]: [−0.047; −0.001], *p*-value: 0.038*) and delta (OR: 0.986, CI: [−0.029;0], *p*-value: 0.051) band power. Moreover, we hypothesized that detected seizures may propagate differently and engage the autonomic central network to a greater extent than undetected seizures, which might involve distinct subcortical pathways. In addition, undetected seizures exhibit a significantly higher increase in delta (*p*-value: 0.0136) and theta band (*p*-value: 0.0236), which has already been described in the literature – reflecting a potential mechanism underlying temporal lobe seizures, which were characterized by impaired consciousness without motor activity (Englot and Blumenfeld, 2009). These seizures were associated with large amplitude slow EEG activity, and neuroimaging signals decreased in the frontal and parietal cortices, contrary to the fast poly-spike activity and high blood flow in limbic structures and subcortical regions. Thus, they proposed the “network inhibition hypothesis,” in which seizure activity propagates to subcortical regions necessary for cortical activation, such as the lateral septum, allowing the cortex to descend into an inhibited state of unconsciousness without motor manifestation during focal secondary generalized seizures (Englot and Blumenfeld, 2009).

Correlation between VENG alterations and ictal HR changes showed that seizures were characterized by bradycardia in most rats. Among the rats studied, 1 out of 6 displayed ictal tachycardia, characterized by a mean average increase of 3 ± 1.6% and an average peak increase of 6.8 ± 2.8% relative to pre-ictal HR values. In 5 out of 6 rats, we observed a mean average HR decrease of 2.1 ± 2.3% and an average peak decrease of 7.6 ± 6% relative to pre-ictal HR values. Binomial logistic regression analysis revealed a correlation between detection and higher HR changes, with the VENG-detected seizure group exhibiting more pronounced alterations. This underscores the meaningful association between ictal VENG changes and cardiac variations, aligning with findings from other studies (Harreby et al., 2011; Naggar et al., 2022).

This study encountered several limitations that warrant acknowledgment. First, the size of the experimental group was insufficient to allow for separation into two distinct groups: a training group for refining detection parameters and a test group for validation. Moreover, the small sample size limits the strength of the conclusions drawn from the logistic regression analyses. Future studies should increase the number of rats to test the algorithm’s performance and obtain a more robust representation of seizure characteristics, thereby reinforcing the results presented in the logistic regression analyses. Alternatively, evaluating algorithm performance in spontaneous recurrent seizures induced by kainic acid could further strengthen the results, highlighting the longitudinal aspect of the kainic acid model. Second, KA injection provokes multiple seizures characterized by a high-frequency onset evolving into spike–wave discharges. Usually, we are able to induce two to five seizures in our animals. However, defining the offset became more and more challenging for later seizures, primarily due to increasing interictal activity. While this does not represent a significant limitation, it may result in a slight variation in seizure duration. This consistency in identifying seizure onset allowed us to accurately determine the delay between EEG seizure onset and VENG seizure onset, ensuring the reliability of our findings, which is a major challenge in the context of seizure detection. Additionally, anesthesia might influence the autonomic modifications during seizures, as it is known in the literature that ketamine decreases parasympathetic tone, homeostatic reflex and vagal activity (Irnaten et al., 2002; José et al., 2022). However, the anesthesia protocol was uniformly applied across all animals in our cohort. Deep anesthesia maintenance was confirmed by the predominant presence of delta waves during the baseline period, constituting the largest proportion of observed waves (54.8 ± 2.6%), followed by theta waves (22.5 ± 6.4%). Finally, a major limitation is the lack of respiration recording, which hinders the direct correlation between ictal VENG modulation and respiratory dysregulation, such as apneas or tachypnea during seizures. While direct respiratory recording was not feasible in our experimental protocol, literature supports indirect estimation of respiratory rate from ECG-derived R peak amplitude modulation (Zhao et al., 2008; Charlton et al., 2018), a method successfully applied previously in our group (Stumpp et al., 2020, 2021). Stump et al. demonstrated synchrony between ECG-derived respiration and VENG signal bursts, indicating potential for correlating these measures to understand respiratory dynamics during seizures (Stumpp et al., 2020, 2021).

Although RNS remains the most reliable detection method and demonstrates high efficacy, due to its highly invasive nature and restriction to focal epilepsy, other closed-loop stimulation strategies should be considered. VNS is applicable to both pediatric and adult populations and is effective for epilepsy of both lesional and non-lesional origin, whether focal or generalized (Toffa et al., 2020). Additionally, VNS improves not only seizure frequency but also comorbidity factors, such as depression (Klinkenberg et al., 2012). However, there is a critical need to enhance seizure detection for closed-loop VNS. Within this context, our research aims to explore alternative methods for seizure detection. This study underscores the potential of VENG recording as a promising alternative for HR-based seizure detection. This investigation primarily adopts a descriptive approach. Future investigations are needed to confirm the applicability of ictal VENG alterations for spontaneous seizure detection in chronic epilepsy models, and determine sensitivity and specificity in these models as well as the comparison between only open loop and combined open and VENG-based closed-loop stimulation. After refining our detection algorithm, expanding the animal cohort will be necessary to gain a more precise understanding of its sensitivity and accuracy. Managing motion constitutes a critical challenge requiring the use of thresholds to reject motion artifacts. Machine learning could assist us in identifying specific patterns in each patient and setting appropriate detection thresholds. This will be addressed in the future, following our investigations in the chronic model. Furthermore, broadening our research to multiple types of seizures is crucial, with the ultimate goal of enhancing the sensitivity of closed-loop vagus nerve stimulation (VNS) devices across all seizure types.

## Data availability statement

The raw data supporting the conclusions of this article will be made available by the authors, without undue reservation.

## Ethics statement

The animal study was approved by Animal Experimental Ethical Committee of UCLouvain University and the Brussels environment committee under the reference 2022/UCL/MD/066. The study was conducted in accordance with the local legislation and institutional requirements.

## Author contributions

EA: Conceptualization, Data curation, Formal analysis, Investigation, Methodology, Resources, Writing – original draft, Writing – review & editing. EG: Methodology, Software, Writing – review & editing. AD: Writing – review & editing. EC: Writing – review & editing. RR: Methodology, Writing – review & editing. AN: Conceptualization, Methodology, Supervision, Writing – review & editing. RT: Conceptualization, Funding acquisition, Methodology, Resources, Supervision, Writing – review & editing.
